# Speculative Review on the Feasibility of Porcine Circovirus 2 Elimination

**DOI:** 10.3390/ani15182744

**Published:** 2025-09-19

**Authors:** Joaquim Segalés, Marina Sibila

**Affiliations:** 1 Departament de Sanitat i Anatomia Animals, Facultat de Veterinària, Universitat Autònoma de Barcelona (UAB), 08193 Bellaterra, Barcelona, Spain; 2 Unitat Mixta d’Investigació IRTA-UAB en Sanitat Animal, Centre de Recerca en Sanitat Animal (CReSA), Universitat Autònoma de Barcelona (UAB), 08193 Bellaterra, Barcelona, Spain; marina.sibila@irta.cat; 3 WOAH Collaborating Centre for the Research and Control of Emerging and Re-Emerging Swine Diseases in Europe (IRTA-CReSA), 08193 Bellaterra, Barcelona, Spain; 4 Institut de Recerca i Tecnologia Agroalimentàries (IRTA), Programa de Sanitat Animal, Centre de Recerca en Sanitat Animal (CReSA), Universitat Autònoma de Barcelona (UAB), 08193 Bellaterra, Barcelona, Spain

**Keywords:** porcine circovirus 2 (PCV2), vaccine, elimination, eradication, whole-herd vaccination

## Abstract

Porcine circovirus 2 (PCV2) is still infecting pigs after almost 20 years of massive vaccination all over the world. Vaccines are highly effective at counteracting the clinical signs of systemic disease caused by PCV2 and can significantly reduce the number of subclinically infected pigs. However, current vaccination programs based on one single dose in piglets are insufficient to prevent infection in a proportion of animals. Moreover, systematic vaccination of the herd changes viral epidemiology and, consequently, can cause modifications in infection timing. Such a scenario may prompt intrauterine and piglet early infections, thus facilitating viral circulation even before vaccination takes place. Considering the demonstrated high vaccine efficacy, it would be legitimate to explore the possibility of eliminating PCV2 from swine herds, but only one attempt to eliminate the virus from a herd has been published so far. The present speculative review evaluates the existing scientific literature regarding the feasibility of getting rid of this virus under commercial farm conditions. The use of PCV2 vaccination in all swine populations within a herd and the implementation of regional or national control programs are foreseen as compulsory for the eventual successful elimination of this endemic viral infection.

## Simple Summary

Porcine circovirus 2 (PCV2) is one of the main pathogens infecting swine. It can cause systemic and reproductive diseases and subclinical infections, and it has been associated with porcine dermatitis and nephropathy syndrome occurrence. Since its discovery during late 1990s, different scenarios have been experienced by swine producers over the world. The impact of a devastating disease with high mortality and lack of control tools was followed by the advent of highly efficacy vaccines. Despite having these products for around 20 years, PCV2 is still present worldwide. The present speculative review tackles those scientific aspects that may be considered for the eventual full elimination of PCV2 from pig farms.

## 1. Introduction

Porcine circovirus 2 (PCV2), a small, non-enveloped, single-stranded DNA virus belonging to the *Circoviridae* family, is one of the most economically significant viral pathogens affecting modern swine production [1]. PCV2 is the etiological agent of porcine circovirus diseases (PCVDs), a group of conditions including systemic disease (PCV2-SD), subclinical infection (PCV2-SI), reproductive disease (PCV2-RD), and porcine dermatitis and nephropathy syndrome (PDNS). Among these, PCV2-SD and PCV2-SI are the most globally widespread and impactful [2].

Since its emergence in the 1990s, PCV2 has achieved an endemic status in nearly all pig-producing regions of the world. Transmission primarily occurs via oro-nasal and fecal–oral routes, but vertical transmission and shedding through various secretions also contribute to its wide dissemination [3]. High-density pig production, continuous flow management, and limited biosecurity in some systems have facilitated its continuous circulation and dissemination. Moreover, PCV2 is a highly resistant virus in the environment and to several disinfectants [4,5], which makes its elimination from the environment a challenging issue. In addition, at least in the absence of vaccination, the virus can persist for long in a proportion of animals, in some cases up to 21 weeks [6]. PCV2 exhibits a high mutation rate for a DNA virus, contributing to generating variability (up to nine genotypes have been proposed so far) and genotype shifts over time [7,8], which contributes to its persistence in the worldwide pig population.

The introduction of PCV2 vaccines in the mid-2000s was a cornerstone in controlling PCVDs. Current commercial vaccines, primarily based on the PCV2a genotype but nowadays with some products containing also PCV2b or PCV2d [9], are safe and highly efficacious at preventing clinical disease and reducing viral load and shedding [10,11]. They are typically administered as a single intramuscular dose to piglets between 3 and 4 weeks of age, around weaning, although alternative strategies exist. Despite the success of these vaccines in mitigating the clinical impact of PCV2, they do not confer sterilizing immunity [9], and infections can still occur, as observed in many field studies comparing vaccinated versus non-vaccinated pigs [12,13,14,15]. Moreover, experimental infections using one dose also showed that a percentage of vaccinated animals display evidence of infection [16,17,18]. These subclinical infections, though not causing overt disease, can impair growth performance [10], leading to substantial economic losses [19].

Given the strong performance of vaccines in preventing disease and reducing transmission, it would be pertinent to consider whether PCV2 elimination could be a realistic objective under commercial farm conditions [20]. To date, only one formal attempt at PCV2 elimination from a swine herd has been reported [21], highlighting the lack of systematic approaches aimed at this goal. The present speculative review explores whether PCV2 elimination at the herd level is feasible through optimized vaccination strategies and whether regional or national coordinated efforts could enhance the chances of success. Importantly, this document does not use the word eradication, as this refers to the complete and permanent global removal of a disease, meaning no cases exist anywhere and no further control measures are needed. In contrast, elimination means reducing a disease to zero cases within a specific geographic area but ongoing interventions are required to prevent its reintroduction [22]. So far, only two diseases have been truly eradicated worldwide, smallpox [23] and rinderpest [24], and such achievements were possible because of the existence of excellent vaccine products against the causative pathogens.

## 2. Diagnosis of PCV2 Infections

PCV2 is regarded as a ubiquitous pathogen, being present in virtually all swine farms and consistently detectable in both animals and the environment [25]. Remarkably, PCV2 DNA has also been identified in facilities devoid of pigs, including warehouses, offices, farm perimeters, and even on farm staff clothing [26]. Intriguingly, the latter study reported that viral loads in non-pig areas were equal to or exceeded those detected in vaccinated pig housing areas. Thus, PCV2—or fragments of its genome—can be found throughout farm environments; however, such detection does not necessarily reflect its impact on herd health. This highlights that real-time quantitative PCR is a powerful tool for detecting PCV2 nucleic acids but cannot be considered diagnostic evidence of PCVDs [2]. It is important to bear in mind that the lack of detection of PCV2 DNA in an individual sample using this technique is not a guarantee of absence, as it may be due to viral loads below the quantification limit of the method.

Vaccinated herds typically exhibit significantly reduced prevalence of infection and viral loads compared to non-vaccinated populations [27], explaining why PCV2-SD or PCV2-RD are rarely diagnosed in vaccinated farms. Importantly, the diagnostic criteria for PCVDs remain unchanged and continue to rely on established parameters [28]: (1) the presence of compatible clinical signs and gross lesions; (2) microscopic lesions in lymphoid tissues (PCV2-SD) or fetal myocardium (PCV2-RD); and (3) demonstration of viral genome or antigen within histological lesions.

What remains unclear, however, is the precise impact of PCV2-SI on herd productivity. Although it is recognized that higher individual viral loads in non-vaccinated pigs are associated with reduced average daily weight gain (ADWG) [29], the quantitative relationship between specific viral loads and production losses has not been fully elucidated. Considering that PCV2-SI likely exerts variable economic impacts across herds [19], the ideal management goal would be to minimize viral circulation to the lowest levels possible, ultimately aiming for elimination of PCV2 infection from swine populations.

## 3. PCV2 Vaccines and Correlates of Protection

Commercially available vaccines are formulated either as inactivated whole-virus preparations (original or PCV1-PCV2 chimeric viruses) or, more commonly, as subunit vaccines containing the viral capsid (Cap) protein [9]. Considering the age of vaccination and other vaccines applied at a similar time, some bivalent products also combine PCV2 and *Mycoplasma hyopneumoniae* antigens to broaden disease control. All of these vaccines have consistently demonstrated excellent efficacy in preventing clinical manifestations of PCV2-SD, as well as significantly reducing viral loads in infected animals.

The immune mechanisms conferring protection against PCV2 involve both humoral and cellular responses [30]. Neutralizing antibodies directed against the viral Cap protein are crucial for limiting viremia and preventing viral dissemination [31,32,33]. However, protection is not solely antibody-mediated. Several studies have highlighted the importance of a robust cell-mediated immune (CMI) response, particularly the activation of PCV2-specific IFN-γ-secreting T cells, in the resolution and control of infection [14,31]. The balance and timing of these immune responses are essential for effective protection, and their magnitude can be influenced by both host and environmental factors [34].

The timing of vaccine administration is a critical determinant of its efficacy. Because most vaccines are applied around weaning, maternally derived immunity (MDI) can interfere with the induction of active immune responses, particularly when antibody titers are very high at the time of piglet vaccination [35]. Although modern PCV2 vaccines are generally effective even in the presence of MDI [36], interference remains a potential limitation under certain conditions, and the optimal vaccination window may vary between farms [37,38].

Moreover, co-infections at the time of vaccination, such as those caused by porcine reproductive and respiratory syndrome virus (PRRSV) or, eventually, swine influenza virus (SIV), can impair vaccine efficacy by modulating the pig’s immune responsiveness [39,40]. Globally speaking, immunosuppressive pathogens and concurrent inflammatory conditions can impair vaccine-induced immune activation, potentially resulting in suboptimal protective responses or delayed development of immunity [41]. These factors must be considered when designing vaccination protocols tailored to specific herd health statuses.

Altogether, understanding the correlates of protection and optimizing vaccine timing are fundamental to maximizing the benefits of PCV2 immunization. These considerations also become particularly relevant when exploring enhanced or alternative strategies aimed at reducing viral transmission and approaching the ambitious goal of PCV2 elimination at the herd level.

## 4. Single and Double PCV2 Vaccination in Piglets

The vast majority of PCV2 vaccination protocols currently implemented in commercial swine production systems rely on a single vaccine dose administered to piglets at weaning [42]. Numerous experimental and field studies have validated the efficacy of this single-dose strategy in reducing clinical disease, lowering viral loads, and improving production parameters, such as ADWG and feed conversion ratio [12,43,44,45]. These benefits have been confirmed under a wide range of epidemiological conditions and herd profiles, which has consolidated single-dose vaccination as the industry standard over the past two decades [9].

However, despite the overall success of single-dose PCV2 vaccination, it has become increasingly evident that this approach does not fully prevent viral infection [18]. A proportion of vaccinated piglets still become subclinically infected, particularly in settings where the timing of natural infection precedes or coincides with the development of vaccine-induced immunity. In this context, interest in alternative immunization strategies, including two-dose (booster) regimens, has gained attention in both experimental and field settings [46,47,48].

Experimental studies evaluating double PCV2 vaccination in piglets—generally involving two doses given two to three weeks apart—have shown enhanced immunological responses, with higher titers of neutralizing antibodies and more robust PCV2-specific T cell activation compared to single-dose counterparts [46,48,49]. These boosted immune responses are considered to be associated with a more effective reduction of viremia and viral shedding, especially in the face of early infection pressure or high maternal antibody interference. Moreover, in an experimental case, double vaccination of piglets resulted in sterilizing immunity upon PCV2 challenge two weeks after the booster [46].

Some studies report better growth performance, lower viral prevalence, and more consistent seroconversion in double-vaccinated groups, even sometimes using split doses [50,51]. Nevertheless, the magnitude of benefit may vary depending on the farm’s baseline PCV2 status, vaccination timing, and concurrent health challenges.

Importantly, while double vaccination can enhance protection in specific circumstances, it also implies increased labor, cost, and handling stress for piglets, which must be balanced against the expected benefits. To date, the systematic application of two-dose schedules is not widespread but may be justified in high-risk herds or in efforts aiming at reducing viral circulation to minimal levels.

In summary, although single-dose PCV2 vaccination remains broadly effective and practical, two-dose regimens may offer added protection in specific epidemiological contexts. These findings are relevant when considering intensified vaccination strategies as part of a broader goal of reducing or potentially eliminating PCV2 infection in commercial swine populations.

## 5. Vaccination of Reproductive Stock

Vaccination of multiparous sows against PCV2 has become an increasingly common practice in many commercial swine systems. While initial vaccination efforts focused mainly on piglets and then in piglets and gilts, the immunization of sows is recognized as an important component of comprehensive PCV2 control [52,53,54], particularly when aiming to reduce viral circulation within herds or to prevent vertical and early horizontal transmission.

Sow vaccination serves two primary objectives. First, it aims to ensure the production of high levels of maternally derived antibodies (MDA) in colostrum, which can provide passive protection to neonatal piglets during their most vulnerable stages. High MDA titers in piglets can delay early PCV2 infection and help bridge the gap until active immunity develops post-vaccination [55,56]. Second, sow vaccination helps to protect gestation itself by minimizing the risk of intrauterine infection. Vertical transmission of PCV2 from dam to fetus has been documented, and fetal infections may lead to reproductive failure, mummification, or suboptimal development in utero. By vaccinating sows, particularly before breeding or during early gestation, such risks can be mitigated, improving both reproductive and piglet productivity outcomes [52,53,54,55,56,57]. Late gestation sow vaccination would not be as advantageous, as high MDI levels in piglets might jeopardize the vaccine’s efficacy in them [35]. However, it has been proposed that intradermal PCV2 vaccination in piglets with high MDA does not interfere with the development of humoral and cellular immune responses [58]. As a practical alternative in sow farms, blanket vaccination approximately every 6 months—the duration of immunity claimed by most commercial vaccines—should ensure the vaccination of all productive sows in the herd. This strategy would help reduce viral circulation at early piglet ages and decreasing the likelihood of vertical infection in fetuses [54].

Gilt vaccination plays a crucial role in acclimatizing replacement animals before their introduction into the sow herd. Gilts often have a different immunological background compared to multiparous sows [56], and naïve or suboptimally immunized gilts can act as amplifiers of viral circulation within the herd. Vaccination of gilts—either during quarantine or prior to first service—not only protects them from clinical disease and reproductive losses but also contributes to maintaining herd-level immunity and consistent MDA transfer to their future offspring [42].

Boar vaccination, although less commonly practiced, is another strategy to consider [59,60]. Although the role of boars in PCV2 epidemiology is less well characterized, it is known that the virus shed by semen of infected boars is not enough to cause venereal transmission [61]. However, the virus can infect boars, and they can shed the virus through multiple routes, contributing to horizontal spread of PCV2 [62]. Therefore, boar vaccination should be of more interest in high-health or nucleus herds.

Overall, the vaccination of reproductive stock enhances both direct protection and herd immunity, and its integration into control strategies is essential when aiming not only to mitigate disease but also to move toward elimination of PCV2 in swine herds. The coordinated immunization of sows, gilts, and potentially boars, when aligned with piglet vaccination and strict biosecurity, constitutes a key pillar of PCV2 infection management.

## 6. PCV2 Mass Vaccination

The concept of mass vaccination is central to infectious disease control and has been successfully employed against a variety of swine pathogens, including Aujeszky’s disease virus (ADV), classical swine fever virus (CSFV), and foot-and-mouth disease virus (FMDV) [63,64,65]. The primary goal of mass vaccination programs is to generate continuous and uniform population immunity that effectively interrupts transmission chains, reducing both infection incidence and pathogen prevalence at the herd or regional level [66]. This strategy not only aims to protect individual animals but also seeks to establish herd immunity thresholds sufficient to suppress or eliminate the pathogen in a defined population. To reach these goals, potent vaccine efficacy together with a sustained administration over time are compulsory.

**Figure 1:**
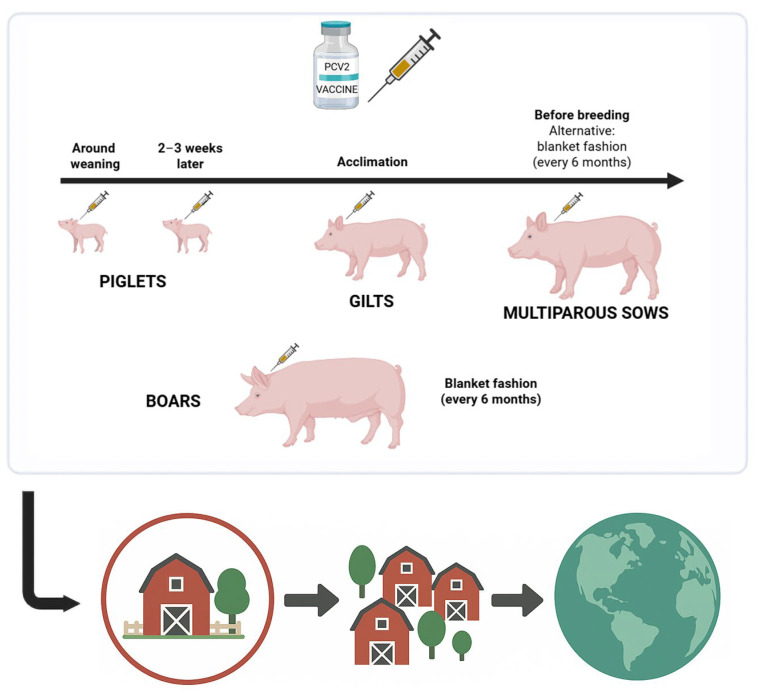
Proposed schedule for mass vaccination with the objective of PCV2 elimination. Mass vaccination programs entail the systematic and sustained immunization of all swine subpopulations within a farm performed at regional, national, and/or global levels (image created with biorrender.com and chatgpt.com).

Applied to PCV2, mass vaccination entails the systematic and sustained immunization of all swine subpopulations—including piglets (ideally with a double dose), gilts (during acclimation), sows, and potentially boars—across production flows (Figure 1). Sow vaccination should be preferably applied before breeding, as it would provide protection against PCV2 infection during gestation; blanket vaccination could also be an option, as it ensures the immunization of the whole breeding herd. In this latter option, a proportion of sows would be vaccinated close to farrowing, which implies the transfer of high levels of MDI to their offspring and putative interference with piglet vaccination. Globally speaking, the expectation is to establish levels of immunity in the entire herd high enough to minimize viral replication and shedding, potentially reducing the force of infection and interrupting transmission cycles. This approach could, theoretically, lead to progressive reduction in PCV2 prevalence and even eventual elimination, particularly if implemented in a coordinated program within regions or countries.

However, the reality of eventual PCV2 elimination through mass vaccination is more nuanced. Despite almost two decades of widespread vaccine use, PCV2 remains endemic in probably all pig-producing regions of the world. Although clinical disease is now rare in vaccinated herds, subclinical infection and viral circulation persist in a significant proportion of populations [2]. Several factors contribute to this, including heterogeneity in vaccine schedules and coverage, single-dose vaccination of piglets, incomplete herd immunity, viral evolution and genotype shifts, and challenges related to MDI interference or early-life infection.

Notably, the only published attempt at PCV2 elimination through mass vaccination under commercial conditions is the study by Feng et al. [21]. In this work, a closed herd underwent whole-population PCV2 vaccination for over one year. The outcome demonstrated that viral detection became increasingly rare, and, after this period, PCV2 was no longer detectable in the serum of piglets, which did not seroconvert at the time of slaughter. However, after stopping this mass vaccination program, the virus was detected again a few months later [21], which suggests that maintenance of the vaccination strategy should be needed to reach continuous non-detectable levels of the virus. Considering that a systematic and continuous program of PCV2 mass vaccination has not been implemented at regional or country levels to date, the Feng et al. [21] study stands alone and highlights the need for further validation and scaling of such mass vaccination approaches.

Lessons from other diseases reinforce that mass vaccination can be an effective route towards pathogen elimination, but success depends on consistent application, high vaccine efficacy, and strict biosecurity. In the case of PCV2, translating the theoretical benefits of mass vaccination into field-level elimination will require coordinated efforts, enhanced diagnostics, and long-term commitment from the swine industry.

## 7. Discussion

The prospect of eliminating PCV2 through mass vaccination protocols is conceptually sound and grounded in precedents from other swine infectious diseases [20]. The demonstrated efficacy of existing PCV2 vaccines in preventing clinical disease and reducing viral load [42] provides a solid foundation for this approach. However, the likelihood of achieving full PCV2 elimination solely through vaccination remains uncertain. Although isolated success has been reported under controlled conditions [21], widespread field application faces several challenges that extend beyond vaccine performance alone.

For mass vaccination to have a meaningful impact on PCV2 circulation, it would need to be comprehensively implemented across all swine populations—including piglets in a double dose, gilts, sows, and potentially boars—in a coordinated and sustained manner. Such an approach should ideally be embedded within broader regional or national control and biosecurity programs, mirroring strategies that have led to the elimination of other viral diseases, such as ADV, FMD, and CSF [67,68]. Key pillars of these programs would include standardized vaccination protocols, continuous monitoring of viral prevalence, and robust biosecurity frameworks to prevent reintroductions.

Despite the biological plausibility, several practical and economic limitations may hinder the large-scale adoption of such strategies [69]. First, the cost of vaccines and labor-intensive administration protocols represent significant investments, particularly for large or resource-limited farms. Very importantly, such cost-effectiveness should overcome the one offered by current vaccination schedules. Moreover, repeated handling of animals for vaccination may raise animal welfare concerns, especially under current paradigms that increasingly value low-stress management practices. In this context, needle-free vaccination technologies, such as intradermal delivery systems, represent a promising avenue for the future. These methods could potentially reduce animal stress, improve safety for handlers, and enhance compliance with mass vaccination protocols, particularly when targeting populations like sows and gilts. Furthermore, advances in vaccine formulations, including longer-lasting immunity and multivalent approaches, may further support the feasibility of virus elimination efforts.

Ultimately, while PCV2 elimination through mass vaccination is biologically feasible, its realization would depend on a multi-layered effort. This includes industry-wide engagement, public–private partnerships, and policy-driven support to incentivize coordinated action. Additionally, continued efforts are necessary to refine vaccination timing and monitor viral evolution. Further limitations include the comparability of monitoring schedules due to variability in diagnostic assays (mainly qPCR), the possible role of wildlife reservoirs, and, most importantly, vaccination and biosecurity compliance in all farms.

A final consideration concerns the genotypes included in vaccines intended for PCV2 elimination. Although multiple PCV2 genotypes have been identified [4], extensive evidence indicates strong cross-protection among them, supporting the notion that PCV2 represents a single serotype [70]. Nevertheless, vaccines formulated with multiple genotypes or with antigens homologous to the predominant strains circulating in a given population may theoretically provide broader epitope coverage [71,72] and potentially enhance protective efficacy. This aspect could be relevant in the context of PCV2 elimination strategies; however, current evidence does not consistently demonstrate that vaccine genotype selection significantly modifies clinical protection outcomes.

## 8. Conclusions

Elimination of PCV2 through vaccination is a scientifically feasible objective strongly supported by the proven efficacy of current immunization tools. Achieving this goal requires comprehensive vaccination of all swine populations alongside coordinated regional or national control programs. Ultimately, we believe PCV2 elimination is possible—it depends not on the pigs but on human commitment, coordination, and sustained effort. However, the practical implementation of PCV2 elimination strategies must be economically justified, as the investment in mass vaccination programs should demonstrate a clear return under current conditions where PCV2-SI is the most prevalent PCVD. Ultimately, the development of novel vaccines with increased potency would also help in an eventual scenario of mass vaccination with the objective of eliminating PCV2.

## Data Availability

Not applicable.
